# Low Back Pain and Associated Factors among Hairdressers in Northern Ethiopia: A Cross-Sectional Study

**DOI:** 10.1155/2021/2408413

**Published:** 2021-04-27

**Authors:** Gebrerufael Solomon Tsegay, Berihu Fisseha Gebremeskel, Selam Desalegn Gezahegn, Desalegn Massa Teklemichael

**Affiliations:** ^1^Department of Physiotherapy, School of Medicine, College of Health Science and Ayder Comprehensive Specialized Hospital, Mekelle University, P.O. Box-1871, Mekelle, Tigray, Ethiopia; ^2^Department of Epidemiology, School of Public Health, College of Health Science, Mekelle University, P.O. Box-1871, Mekelle, Tigray, Ethiopia

## Abstract

Low back pain is one of the major occupational health problems ranked the highest in terms of years lived with disability, and it has an economic burden on individuals and society in general. Hairdressers are high-risk professionals, but they are usually getting less attention in research and policy actions. The objective of this study is to assess the magnitude and associated factors of low back pain among hairdressers working in female beauty salons of Mekelle, Northern Ethiopia. A cross-sectional study was employed from January up to March 2018. A simple random sampling was applied to select 344 participants. Interviewer-administered, pretested, and structured questionnaire was used. Bivariate and multivariate binary logistic regression analyses were performed using SPSS version 23. A *p* value of <0.05 was used to declare statistical significance. A total of 314 hairdressers participated, with a 91.3% response rate. The study found that the prevalence of low back pain was 47.5% with 95% CI (41.8–53.1). Marital status (AOR: 0.40, 95% CI (0.23–0.71)), awkward posture (AOR: 2.59, 95%CI (1.45–4.63)), working greater than 4 days in a week (AOR: 5.12, 95% CI (1.94–13.70)), the task of washing the client's hair (AOR: 4.45, 95% CI (1.93–10.26)), having adjustable washing basin (AOR: 0.35, 95% CI (0.18–0.69)), job stress (AOR: 0.45, 95% CI (0.27–0.77)), and job satisfaction (AOR: 0.58, 95% CI (0.34–0.98)) were factors that show a statistically significant association with low back pain. This study found that low back pain is a major health problem among hairdressers. Marital status, awkward postures, working days per week, the task of washing the client's hair, adjustable washing basin, job stress, and job satisfaction show a strong association with low back pain. The result suggests that effective intervention strategies for low back pain need to include ergonomic improvements and psychosocial and behavioral aspects of the participants.

## 1. Introduction

Low back pain (LBP) is defined as pain, muscle tension, or stiffness on the area between the inferior margin of the 12^th^ rib and inferior gluteal folds with or without sciatica [1]. Back pain can be categorized into specific or nonspecific back pain. Nonspecific back pain is diagnosed when the cause of the pain is unknown and specific back pain refers to a specific cause for the pain [2]. Diagnosis of LBP based on a self-reported questionnaire has been used to estimate the prevalence of LBP in the epidemiological study of community settings [3]. LBP can be acute, subacute, or chronic which affects all ages from children to the elderly and is a very common reason for medical consultations [4].

Work-related low back pain (WRLBP) is one of the common health problems, which most people experience at some point in their life that can cause work absenteeism and occupational disability costs which lead to numerous economic burden on individuals, families, communities, and countries in general with the estimated global point prevalence range from 1.0% to 58.1% and one-year prevalence from 0.8% to 82.5% [1, 5]. Although it was thought of as a problem confined to developed countries, LBP is also a major problem in low- and middle-income countries [6]. A systematic review of recent studies revealed that LBP may be associated with different biomechanical factors (heavy physical work, awkward static and dynamic working postures, and lifting), psychosocial factors (low level of job control, high psychological demands, and work dissatisfaction), and individual factors (younger age, female gender, black or African American race, smoking, high body mass index (BMI), and comorbidity) [7].

Hairdressing is one of the high-risk occupations that sustain low back pain. According to a study in the USA, hairdressers were one of the top 15 high-risk occupations attributed to activities at work in females among 45 major occupations [8]. In the African continent, a study from Nigeria and Egypt shows that LBP was one of the highly prevalent work-related musculoskeletal disorders among hairdressers with an annual prevalence of 76.3% and 12.5%, respectively [9, 10]. In Ethiopia, the LBP accounts for 71.5% of the hairdressers of Addis Ababa [11]. Although the hairdressing salon businesses are growing in number and the professionals are prone to low back pain, epidemiological studies that assess the magnitude of LBP among these workers are limited, and as most of the studies used musculoskeletal disorder as an outcome variable, little is known about the associated factors of LBP among the hairdressers.

Identifying the associated factors is essential for prevention, providing rehabilitation, and professional recommendations on the ergonomic and biomechanical posture for hairdressers. Hence, the main aim of this study is to determine the magnitude and associated factors that contribute to the development of LBP among workers engaged in hairdressing activity in female beauty salons of Northern Ethiopia.

## 2. Materials and Methods

### 2.1. Study Design and Setting

A cross-sectional study was conducted in Mekelle city, Tigray, Northern Ethiopia, from January up to March 2018. Mekelle has a city administration, municipality, and 7 subcities [12]. According to the Tigray Bureau of trade, industry, and urban development office, 451 licensed and registered female beauty salons with a total of 1353 hairdressers were found in the city at the time of data collection.

### 2.2. Study and Source Population

The source populations of the study were all hairdressers working in all-female beauty salons of Mekelle city. All hairdressers who had one-year and above work experience on the job, full-time workers, and working in the sampled female beauty salons of Mekelle city were the study participants. Hairdressers who cannot respond or understand the questionnaire because of health problems and known pregnant women at the time of data collection were excluded from the study.

### 2.3. Sample Size Calculation and Sampling Technique

The sample size for the study was determined using the formula of the single population proportion. Assuming 5% margin of error (d), 95% confidence level (alpha, *α* = 0.05, two-tailed), and prevalence of 71.5% [11], the total sample size is calculated by using the single population formula:

(1) $$\begin{array}{c} n=\frac{{\left(z\left(\alpha /2\right)\right)}^{2}p\left(1-p\right)}{d^{2}},\\ n=313. \end{array}$$

Finally, the sample size (*n* = 344) was obtained by the addition of a 10% nonresponse rate. During the study period, there were a total of 1353 hairdressers working in the 451 female beauty salons with similar characteristics registered by license registration of the Tigray Bureau of trade, industry, and urban development office. Out of the total beauty salons by considering the minimum or one participant from every beauty salon, 344 beauty salons were selected using simple random samplings by a lottery method. Then, samples of 344 eligible hairdressers were selected from each stratum. Individual participants from each beauty salon were selected as follows: one participant was selected from those with one eligible hairdresser, whereas from those with more than one eligible hairdresser, one participant was selected randomly by the lottery method.

### 2.4. Data Collection Tools and Procedures

An interviewer-administered structured questionnaire was used to collect data from the study participants. The questionnaire was divided into four sections. The first section constituted sociodemographic or individual factors, the second section consists of questions about work-related low back pain, the third section constituted questions on ergonomic risk factors, characteristics of the work environment, or work practices, and the fourth section of the questionnaire contains the psychosocial factors of the study participants. The questionnaire to detect the self-reported low back pain was adapted from the Nordic musculoskeletal questionnaire which is a screening and surveillance tool used to analyze musculoskeletal symptoms in an ergonomic or occupational health context [13]. Questioner adapted by the revision of different works of the literature [11, 14–18] was used to assess the sociodemographic, working environment, and psychosocial factors of the study participants.

The generic job satisfaction scale that contains 10 items in which each item had 5-point Likert response categories from strongly agree to strongly disagree was used to assess the job satisfaction of study participants. The validity was tested, and Chronbach's alpha reliability for this scale was 0.77 and has been applied in intensive studies in the literature, including Ethiopia [11, 17, 19, 20]. The score was dichotomized based on reviewed literature as satisfied and dissatisfied with a score of 32–50 and 10–31, respectively [11, 20]. The job stress was assessed by a workplace stress scale that contains 8 items with a 5-point Likert scale response in which the cut-off point was obtained from other kinds of the literature as the total score ≤15/40 indicates that stress is not much of an issue or there is no stress and a total score of 16–40 was considered as they are stressed by their job [11, 18].

### 2.5. Data Quality Control

The questionnaire was first prepared in English and then translated to the local language “Tigrigna” and then back to English to ensure consistency, and a pretest was conducted in 10% of the sampled population. Some modification on the questioners was done and the same understanding in all the data collectors was created. Eight data collectors (physiotherapists and nurses) and two supervisors (physiotherapists) were involved and had taken two days of training before the data collection time about all aspects of the tools, how to approach study participants, and ethical issues. The collected data were checked out for completeness and clarity by the principal investigator and supervisors. Data cleanup and cross-checking were done before analysis.

### 2.6. Study Variables

Dependent variable: low back pain (yes, no)

Independent variables are as follows:

Sociodemographic/individual factors: age, sex, marital status, educational level, monthly income, number of children, BMI, physical activity, and smoking

Working environment and ergonomics factor: professional training, work experience, job category, adjustable chair, adjustable washing basin, workload, work posture, repetitive movement, working hours, and break times

Psychosocial factors: job satisfaction, job stress, and relation to boss or customers

### 2.7. Operational Definition

Low back pain (LBP): having had trouble (ache, pain, and discomfort) in the low back at any time that reduces the activity during the last 12 months [13].

Awkward posture: working with the back bent without support, bending, or twisting of the back or in an awkward way for two or more hours [21].

Body mass index: weight in kilograms divided by the square of the height in meters (kg/m^2^) categorized as underweight = BMI <18.50, normal (healthy) = BMI 18.50–24.99, overweight = BMI 25.00–29.99, and obese = BMI ≥30.00 [22].

Physical activity: exercising any kinds of sports activity at least two times per week with a duration of 30 minutes or at least 150 minutes of moderate-intensity physical activity throughout the week [23].

Static postures: sitting or standing in a restricted space for two or more hours without changing positions [21].

Prolonged standing: standing in a confined working area for two or more hours while performing tasks [24].

Repetitive activity: work involving repeating the same motion with little or no variation every few seconds for two or more hours [21].

### 2.8. Data Analysis

Data were entered, coded, cleaned, and analyzed using the IBM statistical package for social sciences (SPSS) version 23 for Windows in which the mean with its standard deviation and median with its range were determined to summarize numeric variables; similarly, frequencies and proportions of categorical variables were presented using text and tables.

The logistic regression model was used to identify the associated factors with low back pain. Independent variables with a *p* value of less than 0.25 in the bivariate logistic regression were taken into the multivariate logistic regression for controlling the possible main effect of confounders and are reported using CORs. Model fitting was checked using log-likelihood, Hosmer, and Lemeshow goodness of fit test. Finally, variables with *p* < 0.05 in the multivariate analysis were considered statically significant and presented by AOR with 95% C.I.

### 2.9. Ethics Consideration

Ethical clearance was obtained from the ethical review committee of the College of Health Sciences of Mekelle University, and written informed consent was obtained from each of the study participants after being informed in detail about the objective of the study.

## 3. Result

### 3.1. Sociodemographic and Individual Characteristics of Study Participants

A total of 314 hairdressers participated giving a response rate of 91.3%. The majority of the participants were females 289 (92.0%), and the mean age of the study participants was 25.3 ± 4.1 years. Most of them were single (218 (69.4%)) and have no children (223 (71.0%)). Regarding the educational status of the respondents, 183 (58.3%) attended secondary school (9–12) and the median monthly income of the participants was 2000 Ethiopian Birr with a range of 300 to 10,000. Two hundred thirty-nine (76.1%) were served less than 5 years with a median of 3 years, minimum and maximum of 1 and 16 years, respectively. The majority of the participants (284 (79.0%)) had a normal body mass index (BMI). Only 27 (8.6%) of them had physical activity and all of them were nonsmokers (Table 1).

### 3.2. Working Environment and Ergonomic Characteristics of the Study Participants

The majority of hairdressers (280 (89.2%)) were performing their hairdressing activity in a standing position for greater than two hours per day, and 219 (69.7%) worked in an awkward posture to perform their tasks. Out of the participants, 226 (72.0%) worked on the static position for at least 2 hours per day. The majority of the participants (282 (89.8%)) work for more than 4 days per week and had 1 to 5 customers per day with a median number of 5 customers. Hairdressers were asked about the ergonomic situation of furniture at work, 64 (20.4%) were using an adjustable chair, and 241 (76.8%) were using an adjustable washing basin. Two hundred sixty-one (83.1%) of the hairdressers had professional training, of which only 91 (29.0%) had ergonomic education (Table 2).

### 3.3. Psychosocial Characteristics of the Study Participants

Regarding psychosocial factors of the hairdressers, 161 (51.3%) were not satisfied with their current job, while 185 (58.9%) had job stress. About their relationship, 301 (95.9%) reported that they had a good working relationship with their customers. Of the total study participants, 118 (37.6%) had a boss on their work; out of these, 100 (84.7%) had a good working relationship with their boss (Table 3).

### 3.4. Prevalence of Low Back Pain among the Study Participants

Out of 314 hairdressers, the prevalence of LBP was 149 (47.5%) with 95% CI (41.8–53.1). The prevalence of LBP was 57 (59.4%) among the married participants and 121 (55.3%) among those who work in an awkward posture. Similarly, 143 (50.7%) who were working greater than 4 days per week, 136 (50.9%) who participated in the task of washing the client's hair, 103 (42.7%) who used an adjustable washing basin, 106 (57.3%) of those who were stressed in their job, and 95 (59.0%) of those not satisfied on their job developed LBP. Among the participants who had LBP, 108 (72.5%) and 68 (45.6%) of them affected their work and leisure time activities, respectively, and 5 (3.4%) of them changed their job or duties (Table 4).

### 3.5. Factors Associated with Low Back Pain

After the bivariate logistic regression was done for each variable to limit the number of variables and unstable estimates in the final model, variables with a *p* value of less than 0.25 and basic demographic variables in the bivariate were taken into the multivariate analysis. The multivariate binary logistic regression analysis identified that marital status, awkward posture, working day per week, the task of washing the client's hair, adjustable height washing basin, job stress, and job satisfaction showed a statistically significant association with LBP.

Hairdressers who were single were 60% less likely to develop LBP than those who were married (AOR: 0.40, 95% CI (0.23–0.71)). Participants who were working in awkward posture were 2.59 times more likely to develop LBP than those who were not (AOR: 2.59, 95% CI (1.45–4.63)). Participants who work more than 4 days in a week were 5.12 times more likely to develop LBP than those who work less than or equal to 4 days (AOR: 5.12, 95% CI (1.94–13.70)). Hairdressers who work on the task of washing the client's hair were 4.45 times more likely to develop LBP than those who did not work (AOR: 4.45, 95% CI (1.93–10.26)). Respondents who used a washing basin with adjustable height were 65% less likely to develop LBP compared to those who did not use it (AOR: 0.35, 95% CI (0.18–0.69)). Participants who had no job stress were 55% times less likely to develop LBP than those who were stressed (AOR: 0.45, 95% CI (0.27–0.77)). Participants who were satisfied by their job were 42% less likely to develop LBP than those who were not satisfied (AOR: 0.58, 95% CI (0.34–0.98)) (Table 4).

## 4. Discussion

The study was designed to evaluate the prevalence and associated factors of LBP among hairdressers in Mekelle city, Northern Ethiopia. This study found that the prevalence rate of LBP within the past 12 months was 47.5%, which is comparable with studies done in South Africa, Brazil, Greece, and United Kingdom (39.0%–53.0%) [15, 16, 25, 26]. The prevalence was higher compared to the studies conducted in Ibadan Southwest Nigeria, France, and Egypt (12.5%–31.0%) [10, 27, 28]. However, it is lower relative to studies done in Taiwan, Nigeria, and Addis Ababa, Ethiopia (71.5%–83.0%) [9, 11, 29]. The difference might be due to sampling size, the definition of the study area, workload, and the difference in assessment tools of the studies. For example, the study conducted in Egypt that reported a 12.5% prevalence of LBP used a small sample size of 80 hairdressers and 50 office workers [10]. On the other way, hairdressers in Addis Ababa, Ethiopia, showed more prevalence of LBP (71.5%) than this study [11]. It could be due to more sample size or as the participants were from the capital city of the country, they might be open enough to report their pain. In general, the high prevalence of LBP among hairdressers might be due to the stress imposed on the lower vertebral structures by working in prolonged bending and twisting posture. That is why the study conducted in the USA stated that hairdressers were one of the top 15 high-risk occupations for back pain among 45 major occupations [8].

In this study, the majority of the participants which are 72.5% and 45.6% of those with LBP reported that the problem affected their working and leisure time activities, respectively. The United States Bureau of labor statistics found that 53.0% reported taking sick leaves and 10% of them got a permanent work disability pension as a result of back pain [30]. Similarly, studies conducted in Greece, the United Kingdom (UK), and Egypt indicated that LBP was affected in carrying out normal activities and lead to high work absence [10, 15, 25]. This shows that LBP harms the economic, social, and productivity of hairdressers.

In the present study, the marital status shows a statistically significant association in which married hairdressers were more likely to develop LBP than those who were single or unmarried. This might be because married women might have additional tasks in their homes out of the hairdressing job and they might be affected by physical and psychological workload. According to a study done on female employees, married women were more likely to be exposed to harms or pain due to doing house chores, caring for children, and lack of time to spend on relaxation and not doing exercise [31, 32]. However, marital status was not associated with the studies conducted in Ethiopia and Nigeria [11, 28]. This might be due to the difference in sample size and the proportion of married participants among the studies.

This study found that hairdressers working on bending and twisting posture were more likely to develop LBP. This is supported by the study conducted among Egypt and Brazil hairdressers [10, 26]. Incorrect body mechanics during tasks such as bending, turning, and focusing on the task places stress on the highly mobile lumbosacral part of the spine [16]. Bending incorrectly places a mechanical strain on the posterior longitudinal ligament, which results in stress on the facet joints, thereby resulting in LBP [16, 33]. On the contrary, awkward posture was not significantly associated with the musculoskeletal disorder in the study conducted in Addis Ababa, Ethiopia [11]. This might be due to the difference in hairdressing style in different settings and variations in hairdressers' awareness toward comfortable body posture when performing tasks and the availability of modernized ergonomic tools.

In this study, hairdressers who spent more working days in a week (more than 4 days) in hairdressing activity were more likely to develop LBP which is consistent with two studies conducted in Nigeria [9, 28]. However, this was not among the Taiwanese hairdressers [29]. The possible explanation could be as the number of working days increased, the number of customers or workload exceeding this may expose the hairdressers to the risk factors of LBP repeatedly. The study in which the majority of participants worked more days per week indicated that hours spent working per week correlated significantly (*p*=0.002) with how participants bent during their tasks, using their backs rather than their hips and knees [34]. This may result in muscle fatigue, decrease fitness, and increase pain or discomfort in the musculoskeletal system.

Hairdressers who work in the task of washing the client's hair were more likely to develop LBP. It might be due to the fact that the task of washing the client's hair more involves the bending and twisting movements of the spine that can lead to mechanical LBP. This is supported by the study from the USA which reported that the prevalence of back pain increased as the time spent on repeated strenuous physical activities and spent on repeated bending, twisting, or reaching on a typical job is increased [8]. On the other way, hairdressing tasks do not significantly affect discomfort in the body regions of Taiwanese hairdressers [29]. The difference might be the economical difference between the countries that may affect getting the latest and adjustable hairdressing tools.

This study found that hairdressers who used a washing basin with adjustable height were less likely to develop LBP or had a preventing role compared to those who did not use it. Similarly, the incidence of back pain was significantly lower in those hairdressers who used ergonomically adjusted types of equipment [11, 16]. An ergonomically correct washing basin is easily adjustable and maneuverable and allows for easy and regular changes in posture, thereby breaking the chain of a sustained position [16]. The possible explanation may be the fact that when the height is adjustable to the height of hairdressers, the spine will be in an erect position that could decrease the uncomfortable posture of the hairdressers. A study on ergonomic analysis of a hair salon revealed that fixed-height chairs and tables could put hairdressers in uncomfortable positions like twisting and bending to reach different hairdressing materials [33, 34].

Hairdressers who had job stress in this study were more likely to develop LBP, this was supported by the study conducted among France hairdressers, and WHO reports that psychosocial factors appear to play a substantial role in the frequency of LBP [2, 27]. Similarly, participants who were not satisfied with their job were more likely to develop LBP supported by a study result in which low job satisfaction was a risk factor for sickness and absence from work due to LBP [35]. On the other way, not less satisfied workers were more likely to report LBP [36]. Job stress and job dissatisfaction were among the psychosocial demands associated with the increased risk of LBP. It might be because those who were stressed and dissatisfied in their job had different physical influences and workloads that may affect the occurrence of the problem.

This research addressed one of the major occupational health problems among the neglected group of hairdressing workers. However, as it is a 12-month self-reported pain or discomfort, there might be recalled bias; hence, there might be over- and underestimation of the magnitude of LBP. These could lead to a possible variation in the estimation of the association. It is also a cross-sectional study design, in which the temporal occurrence of LBP outcomes cannot be proved and the study also lacks a comparison group. The prevalence rate of LBP is much lower than the assumed rate; this might be due to insufficient sample size. Additionally, since there were fewer male participants, the result of this study might not represent male hairdressers. This study included fewer psychological variables. It is advised in future research projects to make an in-depth analysis by considering postural assessment, objective measurement of ergonomic tools, involving more psychological risk factors, and sufficient sample size.

## 5. Conclusion

This study has shown that low back pain is a common work-related health problem among hairdressers in which marital status, working in awkward postures, more working days per week, the task of washing the client's hair, job stress, and job satisfaction show statistically significant association. Using an adjustable height washing basin has a protective effect on LBP. The study results suggest that effective intervention strategies of LBP have to take into account both ergonomic improvements and behavioral aspects. It is recommended to give attention to promoting health, providing rehabilitation service, and preventing low back pain among hairdressers by giving on-the-job training related to psychological support, comfortable working posture, and utilization of ergonomically designed tools.

## Figures and Tables

**Table 1:** Sociodemographic and individual characteristics associated with LBP among the study participants, April 2018 (*n* = 314).

Variables	Frequency	Percent (%)	LBP (yes)		LBP (no)	
			Frequency	Percent (%)	Frequency	Percent (%)
Gender						
Female	289	92.0	140	48.4	149	51.6
Male	25	8.0	9	36.0	16	64.0
Age group						
≤24	152	48.4	63	41.4	89	58.6
25–29	112	35.7	58	51.8	54	48.2
≥30	50	15.9	28	56.0	22	44.0
Marital status						
Married	96	30.6	57	59.4	39	40.6
Single	218	69.4	92	42.3	126	57.7
Number of children						
No child	223	71.0	98	43.9	125	56.1
1 child	52	16.6	25	48.1	27	51.9
≥2 children	39	12.4	26	66.7	13	33.3
Educational status						
Primary school	89	28.3	48	53.9	41	46.1
Secondary school	183	58.3	78	42.6	105	57.4
Higher education	42	13.4	23	54.8	19	45.2
Work experience (years)						
≤2 years	117	37.3	46	39.3	71	60.7
3–5 years	122	38.9	65	53.3	57	46.7
>5 years	75	23.8	38	50.7	37	49.3
Monthly income (Birr)						
≤1500	133	42.4	52	39.1	81	60.9
1501–3000	106	33.8	62	58.5	44	41.5
>3000	48	15.2	23	47.9	25	52.1
Body mass index (BMI)						
Underweight	39	12.4	11	28.2	28	71.8
Healthy	248	79.0	99	39.9	149	60.1
Overweight	27	8.6	17	63.0	10	37.0
Household activity						
Yes	92	29.3	57	62.0	35	38.0
No	222	70.7	92	41.4	130	58.6
Physical activity						
Yes	27	8.6	7	25.9	20	74.1
No	287	91.4	142	49.5	145	50.5

**Table 2:** Working environment, ergonomic characteristics, and prevalence of LBP among the study participants, April 2018 (*n* = 314).

Variables	Frequency	Percent (%)	LBP (yes)		LBP (no)	
			Frequency	Percent (%)	Frequency	Percent (%)
Prolonged standing						
Yes	280	89.2	140	50.0	140	50.0
No	34	10.8	9	26.5	25	73.5
Static posture						
Yes	226	72.0	107	47.3	119	52.7
No	88	28.0	42	47.7	46	52.3
Awkward posture						
Yes	219	69.7	121	55.3	98	44.7
No	95	30.3	28	29.5	67	70.5
Repeating activity						
Yes	95	30.3	51	53.7	44	46.3
No	219	69.7	98	44.7	121	55.3
Working hours per day						
2–5 hours	46	14.6	13	28.3	33	71.7
6–10 hours	128	40.8	62	48.4	66	51.6
>10 hours	140	44.6	74	52.9	66	47.1
Working days per week						
≤4 days	32	10.2	6	18.7	26	81.3
>4 days	282	89.8	143	50.7	139	49.3
Number of customers per day						
1–5 customers	198	63.1	90	45.5	108	54.5
6–10 customers	116	36.9	59	50.9	57	49.1
Break time in a day						
Yes	40	15.9	23	46.0	27	54.0
No	264	84.1	126	47.7	138	52.3
Adjustable chair						
Yes	64	20.4	32	50.0	32	50.0
No	250	79.6	117	46.8	133	53.2
Adjustable washbasin						
Yes	241	76.8	103	42.7	138	57.3
No	73	23.2	46	63.0	27	37.0
Ergonomics education						
Yes	91	29.0	34	37.4	57	62.6
No	170	54.1	92	54.1	78	45.9
Not professional	53	16.9	23	43.4	30	56.6

**Table 3:** Psychosocial characteristics and prevalence of LBP among the study participants, April 2018 (*n* = 314).

Variables	Frequency	Percent (%)	LBP (yes)		LBP (no)	
			Frequency	Percent (%)	Frequency	Percent (%)
Job stress						
Yes	185	58.9	106	57.3	79	42.7
No	129	41.1	43	33.3	86	66.7
Job satisfaction						
Yes	153	48.7	54	35.3	99	64.7
No	161	51.3	95	59.0	66	41.0
Relationship with boss						
Good	100	31.8	37	37.0	63	63.0
Fair	18	5.8	12	66.7	6	33.3
Have no boss	196	62.4	100	51.0	96	49.0
Relationship with customers						
Good	301	95.9	140	46.5	161	53.5
Fair	13	4.1	7	53.8	6	46.2

**Table 4:** Bivariate and multivariate logistic regression analysis of factors associated with LBP among the study participants, April 2018 (*n* = 314).

Variables	Low back pain		Bivariate	Multivariate	*p* value
	Yes	No	COR (95% CI)	AOR (95% CI)	
Gender					
Female	140 (48.4)	149 (51.6)	1.67 (0.72–3.90)	1.12 (0.28–4.58)	0.870
Male	9 (36.0)	16 (64.0)	1.00		
Age group					
≤24	63 (41.4)	89 (58.6)	0.56 (0.29–1.06)	0.84 (0.29–2.40)	0.738
25–29	58 (51.8)	54 (48.2)	0.84 (0.43–1.65)	0.81 (0.31–2.11)	0.672
≥30	28 (56.0)	22 (44.0)	1.00	1.00	
Marital status					
Married	57 (59.4)	39 (40.6)	1.00	1.00	
Single	92 (42.3)	126 (57.7)	0.50 (0.30–0.81)^*∗*^	0.40 (0.23–0.71)^*∗∗*^	0.002
Number of children					
No child	98 (43.9)	125 (56.1)	1.00	1.00	
1 child	25 (48.1)	27 (51.9)	1.18 (0.65–2.16)	0.68 (0.25–1.88)	0.461
≥2 children	26 (66.7)	13 (33.3)	2.55 (1.25–5.22)^*∗*^	1.43 (0.44–4.64)	0.550
Educational status					
Primary school(1–8)	48 (53.9)	41 (46.1)	0.97 (0.46–2.02)		
Secondary school(9–12)	78 (42.6)	105 (57.4)	0.61 (0.31–1.21)		
Higher education	23 (54.8)	19 (45.2)	1.00		
Work experience					
≤2 years	46 (39.3)	71 (60.7)	1.00	1.00	
3–5 years	65 (53.3)	57 (46.7)	1.76 (1.05–2.94)^*∗*^	1.82 (0.99–3.34)	0.055
>5 years	38 (50.7)	37 (49.3)	1.59 (0.88–2.85)	1.18 (0.57–2.45)	0.649
Monthly income (Birr)					
≤1500	52 (39.1)	81 (60.9)	0.72 (0.38–1.39)	1.61 (0.90–2.90)	0.110
1501–3000	62 (58.5)	44 (41.5)	1.53 (0.77–3.04)	1.64 (0.26–10.24)	0.596
>3000	23 (47.9)	25 (52.1)	1.00	1.00	
Body mass index (BMI)					
Underweight	11 (28.2)	28 (71.8)	0.53 (0.20–1.44)	0.68 (0.30–1.56)	0.365
Healthy	99 (39.9)	149 (60.1)	0.60 (0.27–1.35)	1.20 (0.35–4.07)	0.777
Overweight	17 (63.0)	10 (37.0)	1.00	1.00	
Household activity					
Yes	57 (62.0)	35 (38.0)	2.30 (1.40–3.79)^*∗*^	1.56 (0.85–2.86)	0.150
No	92 (41.4)	130 (58.6)	1.00	1.00	
Physical activity					
Yes	7 (25.9)	20 (74.1)	0.36 (0.15–0.87)^*∗*^	1.85 (0.66–5.20)	0.241
No	142 (49.5)	145 (50.5)	1.00	1.00	
Prolonged standing					
Yes	140 (50.0)	140 (50.0)	2.78 (1.25–6.16)^*∗*^	1.02 (0.35–2.99)	0.977
No	9 (26.5)	25 (73.5)	1.00	1.00	
Static posture					
Yes	107 (47.3)	119 (52.7)	1.02 (0.62–1.66)		
No	42 (47.7)	46 (52.3)	1.00		
Awkward posture					
Yes	121 (55.3)	98 (44.7)	2.95 (1.76–4.94)^*∗*^	2.59 (1.45–4.63)^*∗∗*^	0.001
No	28 (29.5)	67 (70.5)	1.00	1.00	
Repeating activity					
Yes	51 (53.7)	44 (46.3)	0.70 (0.43–1.13)		
No	98 (44.7)	121 (55.3)	1.00		
Working hours per day					
2–5 hours	13 (28.3)	33 (71.7)	1.00	1.00	
6–10 hours	62 (48.4)	66 (51.6)	2.39 (1.15–4.95)^*∗*^	1.71 (0.68–4.29)	0.256
>10 hours	74 (52.9)	66 (47.1)	2.85 (1.38–5.86)^*∗*^	1.50 (0.58–3.85)	0.404
Working days per week					
≤4 days	6 (18.7)	26 (81.3)	1.00	1.00	
>4 days	143 (50.7)	139 (49.3)	4.46 (1.78–11.16)^*∗*^	5.12 (1.94–13.70)^*∗∗*^	0.001
Customers per day					
1–5 customers	90 (45.5)	108 (54.5)	1.00		
6–10 customers	59 (50.9)	57 (49.1)	0.81 (0.51–1.27)		
Break time in a day					
Yes	23 (46.0)	27 (54.0)	1.00		
No	126 (47.7)	138 (52.3)	0.93 (0.51–1.71)		
Hair washing					
Yes	136 (50.9)	131 (49.1)	2.72 (1.37–5.37)^*∗*^	4.45 (1.93–10.26)^*∗∗*^	<0.001
No	13 (27.7)	34 (72.3)	1.00	1.00	
Adjustable chair					
Yes	32 (50.0)	32 (50.0)	1.00		
No	117 (46.8)	133 (53.2)	0.88 (0.51–1.52)		
Adjustable washing basin					
Yes	103 (42.7)	138 (57.3)	0.44 (0.26–0.75)^*∗*^	0.35 (0.18–0.69)^*∗∗*^	0.003
No	46 (63.0)	27 (37.0)	1.00	1.00	
Training of ergonomics					
No	92 (54.1)	78 (45.9)	0.65 (0.35–1.21)	0.68 (0.36–1.28)	0.230
Yes	34 (37.4)	57 (62.6)	0.51 (0.30–0.85)^*∗*^	0.61 (0.28–1.33)	0.214
Not professional	23 (43.4)	30 (56.6)	1.00	1.00	
Job stress					
Yes	106 (57.3)	79 (42.7)	1.00	1.00	
No	43 (33.3)	86 (66.7)	0.37 (0.23–0.60)^*∗*^	0.45 (0.27–0.77)^*∗∗*^	0.004
Job satisfaction					
Yes	54 (35.3)	99 (64.7)	0.38 (0.24–0.60)^*∗*^	0.58 (0.34–0.98)^*∗∗*^	0.043
No	95 (59.0)	66 (41.0)	1.00	1.00	
Relationship with boss					
No boss	100 (51.0)	96 (49.0)	1.00	1.00	
Good	37 (37.0)	63 (63.0)	0.56 (0.34–0.92)^*∗*^	0.62 (0.10–3.76)	0.602
Fair	12 (66.7)	6 (33.3)	1.92 (0.69–5.32)	0.77 (0.36–1.63)	0.496
Relationship with customer					
Good	140 (46.5)	161 (53.5)	1.00		
Fair	7 (53.8)	6 (46.2)	0.39 (0.12–1.28)		

^*∗*^Significant association (bivariate); ^*∗∗*^significant association (multivariate). COR = crude odds ratio, AOR = adjusted odds ratio, and 1.00 = references.

## Data Availability

Data will be available upon request to the corresponding author.

## Abbreviations

AOR: Adjusted odds ratio
BMI: Body mass index
CI: Confidence interval
COR: Crude odds ratio
DALYs: Disability-adjusted living years
LBP: Low back pain
MSD: Musculoskeletal disorder
RBTR: Repeated bending, twisting, or reaching
SPSS: Statistical Package for Social Sciences
WHO: World Health Organization
WRLBP: Work-related low back pain
WRMSD: Work-related musculoskeletal disorder
YLDs: Years lived with disability.

## Supplementary Materials

Supplementary Materials A summary of the STROBE Statement—checklist of items that should be included in reports of cross-sectional studies.
